# Antimicrobial resistance including Extended Spectrum Beta Lactamases (ESBL) among *E*. *coli* isolated from kenyan children at hospital discharge

**DOI:** 10.1371/journal.pntd.0010283

**Published:** 2022-03-31

**Authors:** Stephanie N. Tornberg-Belanger, Doreen Rwigi, Michael Mugo, Lynnete Kitheka, Nancy Onamu, Derrick Ounga, Mame M. Diakhate, Hannah E. Atlas, Anna Wald, R. Scott McClelland, Olusegun O. Soge, Kirkby D. Tickell, Samuel Kariuki, Benson O. Singa, Judd L. Walson, Patricia B. Pavlinac

**Affiliations:** 1 Department of Epidemiology, University of Washington, Seattle, Washington, United States of America; 2 Kenya Medical Research Institute (KEMRI), Nairobi, Kenya; 3 Centre for Microbiology Research, Kenya Medical Research Institute (KEMRI), Nairobi, Kenya; 4 Department of Global Health, University of Washington, Seattle, Washington, United States of America; 5 Department of Medicine, Division of Allergy and Infectious Diseases, University of Washington, Seattle, Washington, United States of America; 6 Department of Laboratory Medicine and Pathology, University of Washington, Seattle, Washington, United States of America; 7 Vaccine and Infectious Disease Division, Fred Hutchinson Cancer Research Center, Seattle, Washington, United States of America; 8 The Childhood Acute Illness & Nutrition (CHAIN) Network, Nairobi, Kenya; 9 Department of Medicine (Allergy and Infectious Diseases), University of Washington, Seattle, Washington, United States of America; 10 Department of Pediatrics, University of Washington, Seattle, Washington, United States of America; Lowell General Hospital, UNITED STATES

## Abstract

**Background:**

Children who have been discharged from hospital in sub-Saharan Africa remain at substantial risk of mortality in the post-discharge period. Antimicrobial resistance (**AMR**) may be an important factor. We sought to determine the prevalence and risk factors associated with AMR in commensal *Escherichia coli* **(*E*. *coli*)** from Kenyan children at the time of discharge.

**Methodology/Principle findings:**

Fecal samples were collected from 406 children aged 1–59 months in western Kenya at the time of discharge from hospital and cultured for *E*. *coli*. Susceptibility to ampicillin, ceftriaxone, cefotaxime, ceftazidime, cefoxitin, imipenem, ciprofloxacin, gentamicin, combined amoxicillin/clavulanic acid, trimethoprim-sulfamethoxazole, azithromycin, and chloramphenicol was determined by disc diffusion according to guidelines from the Clinical and Laboratory Standards Institute (CLSI). Poisson regression was used to determine associations between participant characteristics and the presence of extended-spectrum beta-lactamases (**ESBL**) producing *E*. *coli*. Non-susceptibility to ampicillin (95%), gentamicin (44%), ceftriaxone (46%), and the presence of ESBL (44%) was high. Receipt of antibiotics during the hospitalization was associated with the presence of ESBL (aPR = 2.23; 95% CI: 1.29–3.83) as was being hospitalized within the prior year (aPR = 1.32 [1.07–1.69]). Open defecation (aPR = 2.02; 95% CI: 1.39–2.94), having a toilet shared with other households (aPR = 1.49; 95% CI: 1.17–1.89), and being female (aPR = 1.42; 95% CI: 1.15–1.76) were associated with carriage of ESBL *E*. *coli*

**Conclusions/Significance:**

AMR is common among isolates of *E*. *coli* from children at hospital discharge in Kenya, including nearly half having detectable ESBL.

## Author summary

Children who have been hospitalized in sub-Saharan Africa remain at a high risk of death and morbidity for at least 6 months following discharge. These children may harbor AMR in commensal bacteria following hospitalization, which may be associated with poor outcomes. There are limited data describing AMR and risk factors that are associated with AMR carriage at hospital discharge. In this cross-sectional study of Kenyan children under 5 years of age discharged from hospitals, we found AMR to be high. Children who received antibiotics in the hospital, had limited access to improved sanitation, and who were female had the highest prevalence of ESBL-producing *E*. *coli*.

## Introduction

In most low- and middle-income countries, infectious diseases account for approximately 40% of child deaths [1]. Children living in these settings are regularly exposed to bacterial pathogens from poor sanitation, crowding, and immune-deficiencies, leading to frequent bacterial infections that require antibiotic therapy [2,3]. Children in Sub-Saharan Africa **(SSA)** are at highest risk of death from infection, where limited antibiotic options and a lack of systematic surveillance of antimicrobial resistance **(AMR)** further complicate management decisions [2,4–7].

Children living in SSA who have been discharged from an inpatient hospital stay are at particularly high risk of mortality in SSA [8–11]. For example, post-discharge mortality rates in the year following a hospital stay are often similar to inpatient mortality and 8–9 times higher than mortality rates in age-matched community children [8,12,13]. The mechanisms driving this increased mortality are not clearly understood, though AMR may be a contributing factor [14]. AMR may lead to treatment failure in the post-discharge period and transmissible AMR genes may pose future health risks both to these children and to their communities.

AMR in commensal *Escherichia coli* ***(E*. *coli)*** may act as a surrogate marker for resistance in other pathogenic bacteria in the gut [15–17]. Carriage of AMR in commensal *E*. *coli* has also been shown to be a risk factor for the acquisition of resistant *E*. *coli* infections [15,18–20]. Few studies have quantified AMR in commensal *E*. *coli* in children in SSA, and fewer have characterized AMR in *E*. *coli* at hospital discharge [21]. We conducted a cross-sectional analysis to determine the prevalence of resistance to commonly used antibiotics in commensal *E*. *coli* isolated from Kenyan children discharged from hospital. In addition, we report risk factors for extended-spectrum beta-lactamase (**ESBL**) producing bacterial isolates, an indicator of broad resistance to commonly used beta-lactam antibiotics [7,22–24].

## Methods

### Ethics statement

The study was approved by the Institutional Review Boards of the Kenya Medical Research Institute (SERU 3086), the Kenyan Pharmacy and Poisons Board (Ref. No. PPB/ECCT/15/10/04), and the University of Washington (IRB# 49120). The parent trial was registered at ClinicalTrials.gov (Identifier: NCT02414399). Caregivers provided informed written consent in their preferred language (English, Kiswahili, Kisii, or Luo). If a caregiver was not literate, information was read in the language of their choice and consent was obtained using a witnessed thumbprint.

### Study design

This cross-sectional nested study utilized the antimicrobial susceptibility patterns of *E*. *coli* isolated from children enrolled at hospital discharge as well as clinical, sociodemographic, and child health history information collected during interviews with the primary caregiver and physical exams collected during a recently completed clinical trial. The protocol of the parent trial has been published [25].

### Parent trial

#### Population

Children aged 1–59 months were enrolled at the time of hospital discharge at Kisii and Homa Bay hospitals in western Kenya to assess whether a 5-day course of azithromycin reduces rehospitalization and/or death in the subsequent 6-month period [25]. Eligible children were those who weighed at least 2kg, had been hospitalized and subsequently discharged, planned to remain in the area for at least 6 months, did not have a contraindication to azithromycin, and were not prescribed other macrolide antibiotics [25]. Children were excluded from the study if their hospital admission was solely due to trauma, poisoning, or obvious congenital disorder, if a twin of the same sex was enrolled in the study the same day, or if a legal guardian did not provide consent.

### Data collection

After providing informed consent, caregivers were interviewed by study staff to gather sociodemographic information and relevant medical history using a standardized questionnaire. Diagnoses, procedures, and other relevant medical information were extracted from medical records. Enrolled children underwent a physical examination by a study clinician, which included anthropometric measurements (height (or length if < 24 months), weight and middle upper arm circumference (MUAC)). Weight-for-length/height z-score **(WLZ/WHZ)**, weight-for-age z-score **(WAZ)**, length/height-for-age z-score **(LAZ/HAZ)** were determined using the WHO’s anthropometric macro code for Stata [26].

### Collection and transportation of fecal samples and *E*. *coli* culture

During enrolment in the parent trial, whole stool (or rectal swabs if whole stool was unavailable) was collected from all children. Samples were placed in Cary-Blair media to maintain bacterial integrity during transport for microbiological culture within 24 hours. Briefly, swabs or a sample of stool were streaked onto Mueller-Hinton (**MH**), MacConkey (**MAC**), and eosin methylene blue agars (**EMB**) and incubated in ambient air at 37°C for 18–24 hours. Results from differential media (MAC and EMB) were recorded and the isolation of *E*. *coli* was confirmed using the API 20E system (bioMérieux, Inc) and oxidase reactions as described previously [27]. Confirmed *E*. *coli* isolates were suspended in tryptic soy broth with 15% glycerol and frozen at -80°C. A random subset of enrolled children were chosen to participate in an AMR sub-study. A random number generator from Microsoft Excel was assigned to each child’s patient identification (PID) number from the two main recruiting sites (Kisii and Homa Bay) and PIDs corresponding to random numbers of 0 and 5 were selected for this sub-study. In April 2019, the random selection changed to the selection of child PIDs with caregivers enrolled in an AMR study, whose selection was also based on a random number generator in excel. This switch in random-selection methods occurred due to a secondary aim added to the parent trial to evaluate resistance patterns in both children and their caregivers. *E*. *coli* isolates of selected children were subjected to Antimicrobial Susceptibility Testing (**AST**) using disc diffusion following methods described by the Clinical and Laboratory Standards Institute (**CLSI**) [28]. Isolates were thawed, and quadrant-streaked for isolation onto MAC and incubated at 37°C in ambient air. If more than one colony morphology was seen following restoration on MAC agar, isolates from each distinct morphology (up to 3) were separately subjected to AST testing. Having any *E*. *coli* isolates with non-susceptibility or ESBL-production was considered as indicative of AMR in the child.

### Antibiotic susceptibility testing

The overnight cultures of *E*. *coli* isolates were placed into 5ml of sterile normal saline and adjusted to be uniform with a 0.5 MacFarland standard. Bacterial diluents were used to homogenously cover the surfaces of MH agar using disposable sterile swabs. Prior to incubation, antibiotic discs were added on top of the inoculated MHA using a disc dispenser, ensuring appropriate spacing between discs. The plates were placed in an incubator at 37°C in ambient air for 18–24 hours. Antibiotics tested included ampicillin, ceftriaxone, cefotaxime, ceftazidime, cefoxitin, and imipenem; ciprofloxacin; gentamicin; amoxicillin/clavulanic acid; trimethoprim-sulfamethoxazole; azithromycin; and chloramphenicol. Zone diameters, measured in millimeters, established by CLSI-2020 M-100 were used to determine susceptibility, resistance, or an intermediate designation [28]. Both intermediate and resistant isolates were classified as resistant [28]. As there are no established zone diameters for azithromycin in *E*. *coli*, we used standards for *Salmonella* enterica serovar Typhi [28].

### Determination of ESBL-producing *E*. *coli*

ESBL-production was determined using the double-disc diffusion test, which tests for ceftazidime and cefotaxime with and without clavulanic acid as previously described [29,30]. Quality control was assured by appropriate differences in zone sizes between the two selected antibiotics with and without clavulanic acid for ESBL-producing (NCTC 13351) and ESBL-negative (ATCC 25922) strains of *E*. *coli*. Specifically, suitable performance of the test the ESBL-negative QC strain of *E*. *coli* was confirmed if the difference in the zone size between ceftazidime and the zone size of ceftazidime with clavulanic acid was <5mm and if the difference in the zone size between cefotaxime and the zone size of cefotaxime with clavulanic acid was <5mm. Suitable performance of the test for the ESBL-producing QC strain of E. coli was confirmed if the difference in the zone size between ceftazidime and the zone size of ceftazidime with clavulanic acid was ≥5mm and if the difference in the zone size between cefotaxime and the zone size of cefotaxime with clavulanic acid was ≥5mm.

### Nested study

This nested study included children who participated in the AMR sub-study of the parent trial and from whom *E*. *coli* were isolated at the enrollment visit.

### Statistical analysis

The prevalence of non-susceptibility to each antibiotic of interest was determined and confidence intervals (95%) calculated assuming a binomial distribution. Univariate Poisson regression using robust standard errors was used to determine risk factors for ESBL producing *E*. *coli*. Correlates of interest chosen a priori included child age (in months), HIV infection or exposure, length of hospitalization and antibiotic use in hospital, WAZ, LAZ/HAZ, WLZ/WHZ, enrollment hospital, household toilet type, water source and treatment, household crowding, and ownership of livestock (defined as cows, goats, sheep, pigs and/or chickens). Being referred from another hospital facility and having a hospitalization in the year prior to the one immediately preceding discharge were also examined as risk factors. Having an improved water source was determined by the reporting of water piped to the home or community, a borehole or tube well, spring water through a pipeline, a lined well with a pump or bucket or bottled water as a primary source of water. Household crowding was defined as >2 individuals to a room. Variables were chosen based on their association with AMR in previously published literature or due to clinical and biological plausibility.

Multivariate Poisson regression models with robust standard errors were used to identify independent risk factors for the presence of ESBL producing *E*. *coli* at the time of discharge from hospital. Age, sex, and enrollment hospital were added to multivariable models as were variables that were found to be associated, at the alpha of .05 level, with the resistance outcome in univariate analyses. A separate analysis was conducted to determine whether resistance was associated with the type of sample provided, either whole stool or rectal swabs, to determine whether there may be differences depending on the sample type (S1 Table). Multivariate Poisson regression models adjusting only for variables chosen a priori (age, sex, and hospital) were performed separately (S2 Table). Associations were considered statistically significant at an alpha of 0.05. Analyses were performed in Stata 14 (StataCorp, College Station, Texas).

## Results

### Study population

**Fig 1:**
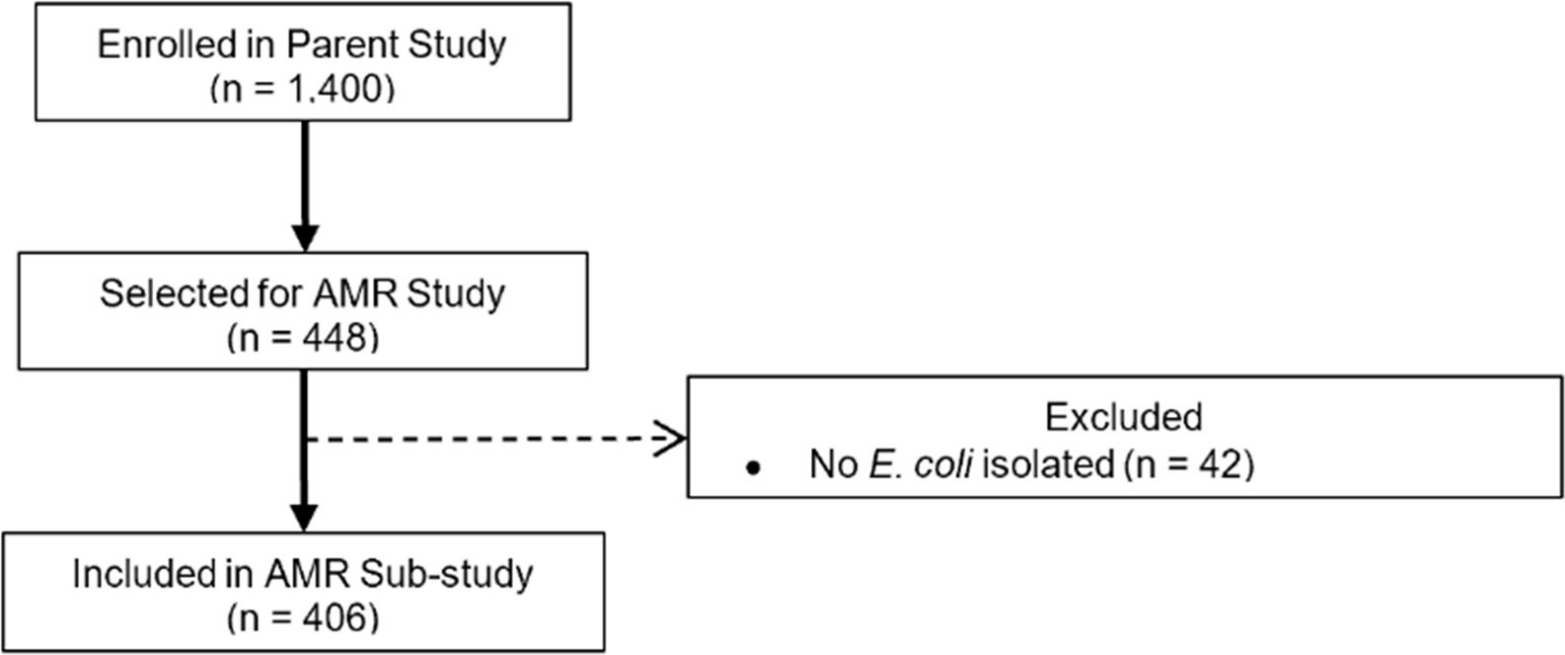
Participant flow chart.

Children were enrolled in the parent trial between June 2016 and November 2019. Among the 448 children randomly selected to have AST performed, *E*. *coli* were identified in the fecal sample from 406 (90.6%). (Fig 1). The median age of the 406 included children was 19 months (interquartile range [IQR] 10–33 months), about 59.4% were male, 2.1% were HIV-infected, and 11% were HIV exposed, uninfected (HEU) (Table 1). During their hospitalization, most children received antibiotics (87.2%); in the subset of children who received antibiotics, the most common were penicillins (82.6%), ceftriaxone (71.6%), and gentamicin (62%). The most frequent diagnoses at admission and discharge were pneumonia, diarrhea, anemia, and malaria. The median length of hospital stay was 3 days (IQR 2–5 days). Children typically came from homes with improved water (84%) and pit latrines (87.9%), although more than half shared toilet facilities with another household. Almost half of the children lived in homes with more than 2 people to a room and most caregivers reported owning livestock (70.6%). The random selection of participants resulted in few significant differences in characteristics between children randomized to this study (S3 Table) or between children with and without *E*. *coli* isolated (S4 Table).

**Table 1:** Descriptive Characteristics.

	Total n	(n = 406) (%)	Kisii n	(n = 242) (%)	Homa Bay n	(n = 164) (%)
**Participant Characteristics**						
Sex						
Male Female	241 165	(59.4%) (40.6%)	143 99	(59.1%) (40.9%)	97 66	(59.5%) (40.5%)
Age (months)						
24 and over 12–23 6–11 1–5	160 121 80 44	(39.5%) (29.9%) (19.8%) (10.9%)	93 70 50 29	(38.4%) (28.9%) (20.7%) (12.0%)	67 51 30 15	(41.1%) (31.3%) (18.4%) (9.2%)
Breastfeeding^i^ Exclusively Breastfed Partially Breastfed Never Breastfed	184 195 5	(47.9%) (50.8%) (1.3%)	74 146 2	(33.3%) (65.8%) (0.9%)	110 49 3	(67.9%) (30.2%) (1.9%)
HIV Status^ii^						
HIV Uninfected HIV Uninfected, Exposed HIV Infected	339 43 8	(86.9%) (11.0%) (2.1%)	218 13 3	(93.2%) (5.6%) (1.3%)	121 30 5	(77.6%) (19.2%) (3.2%)
Nutritional Characteristics^iii^ Neither Stunted nor Wasted Wasted, not Stunted Stunted, not Wasted Stunted and Wasted	271 34 89 11	(66.9%) (8.4%) (22.0%) (2.7%)	166 21 51 4	(68.6%) (8.7%) (21.1%) (1.7%)	105 13 38 7	(64.4%) (8.0%) (23.3%) (4.3%)
**Hospitalization Information**						
Referred from another Heath Facility	106	(26.1%)	72	(29.8%)	34	(20.7%)
Hospitalized ≤1 Year Prior to this Hospitalization^iv^	84	(20.8%)	51	(21.2%)	33	(20.3%)
Length of Hospital Stay (in days)^v^^vi^	3	(2–5)	3	(2–5)	4	(2–6)
Received Antibiotic during Hospitalization	354	(87.2%)	229	(94.6%)	125	(76.2%)
Antibiotic Received during Hospitalization^vii^ Penicillins Ceftriaxone Gentamicin Other	247 131 219 52	(82.6%) (71.6%) (62.0%) (14.7%)	182 70 168 29	(79.5%) (84.3%) (73.4%) (12.7%)	65 61 51 23	(52.4%) (61.0%) (41.1%) (18.6%)
Admitting Diagnosis^viii^ Anemia Gastroenteritis/Diarrhea Malaria Meningitis Pneumonia/LRTI Sickle Cell Sepsis Tuberculosis URTI Other	82 82 191 41 146 43 13 11 28 15	(20.3%) (20.3%) (47.2%) (10.1%) (36.1%) (10.6%) (3.2%) (2.7%) (6.9%) (3.7%)	29 49 101 29 105 10 2 5 21 11	(12.0%) (20.3%) (47.7%) (12.0%) (43.4%) (4.1%) (0.8%) (2.1%) (8.7%) (4.6%)	53 33 90 12 41 33 11 6 7 4	(32.5%) (20.3%) (55.2%) (7.4%) (25.2%) (20.3%) (6.8%) (3.7%) (4.3%) (2.5%)
Prescribed Antibiotic at Discharge	242	(59.8%)	163	(67.4%)	79	(31.7%)
**Household Information**						
Crowding (>2 people/room)	192	(47.4%)	91	(37.6%)	101	(62.0%)
Livestock Ownership	286	(70.6%)	161	(66.5%)	125	(76.7%)
Improved Water Source [31]	340	(84.0%)	217	(89.7%)	123	(75.5%)
Treated Drinking Water^ix^	200	(49.9%)	82	(34.3%)	118	(72.8%)
Shared Toilet^x^	200	(49.4%)	114	(47.1%)	86	(52.8%)
Toilet Type Flushing Pit Latrine Open Defecation	29 356 20	(7.2%) (87.9%) (4.9%)	23 219 0	(9.5%) (90.5%) (0%)	6 137 20	(3.7%) (84.1%) (12.3%)

^i^Of those with data available (n = 384) Current breastfeeding for children ≤6 months; breastfeeding practiced when children were under 6 months; n = 21 unknown

^ii^Uninfected, Exposure Status unknown (n = 11), Exposed, infection status unknown (n = 4); Column percentages of children with exposure and infection status known (n = 390)

^iii^Wasted is defined as WHZ < -2 or MUAC <11.5cm while Stunted is determined by HAZ <-2; MUAC is only taken into consideration in children 6 months or older

^iv^Of the 404 with previous hospitalizations, if any, known

^v^Median and interquartile range provided

^vi^Of those with admission and discharge dates both available (n = 401)

^vii^Not mutually exclusive. Total n = 354 (87.2%) received antibiotics, column percentages are of these children. Other antibiotics given: azithromycin (n = 4), cefuroxime (n = 5), trimethoprim-cotrimoxazole (n = 10), chloramphenicol (n = 16), ciprofloxacin (n = 1), clarithromycin (n = 5), erythromycin (n = 1), tetracycline (n = 1), metronidazole (n = 18)

^viii^Not mutually exclusive. Other diagnoses at admission include: HIV (n = 2), UTI (n = 2), tuberculosis (n = 5), sepsis (n = 9), Poisoning/herbal toxicity (n = 3), asthma (n = 8), upper respiratory tract infection (n = 31), unknown (n = 15)

^ix^Among those who did not report use of bottled water and responded using filters, boiling, or chlorinating drinking water (n = 401)

^x^Shared Toilets are those used by more than 1 household and did not include open defecation (n = 20 all in Homa Bay) and excluding those who did not answer (n = 3)

Participants were included from Kisii Teaching & Referral Hospital (59.7%) and Homa Bay County Referral Hospital (40.3%). Children enrolled in Kisii were more likely to receive antibiotics during hospitalization (94.6%) compared to children enrolled in Homa Bay hospital (76.1%). Children enrolled at Kisii Teaching & Referral Hospital were also less likely to be HIV-exposed or infected or live in a household with a shared toilet, that was crowded, and that owned livestock (Table 1).

### Burden of AMR *E*. *coli*

**Fig 2:**
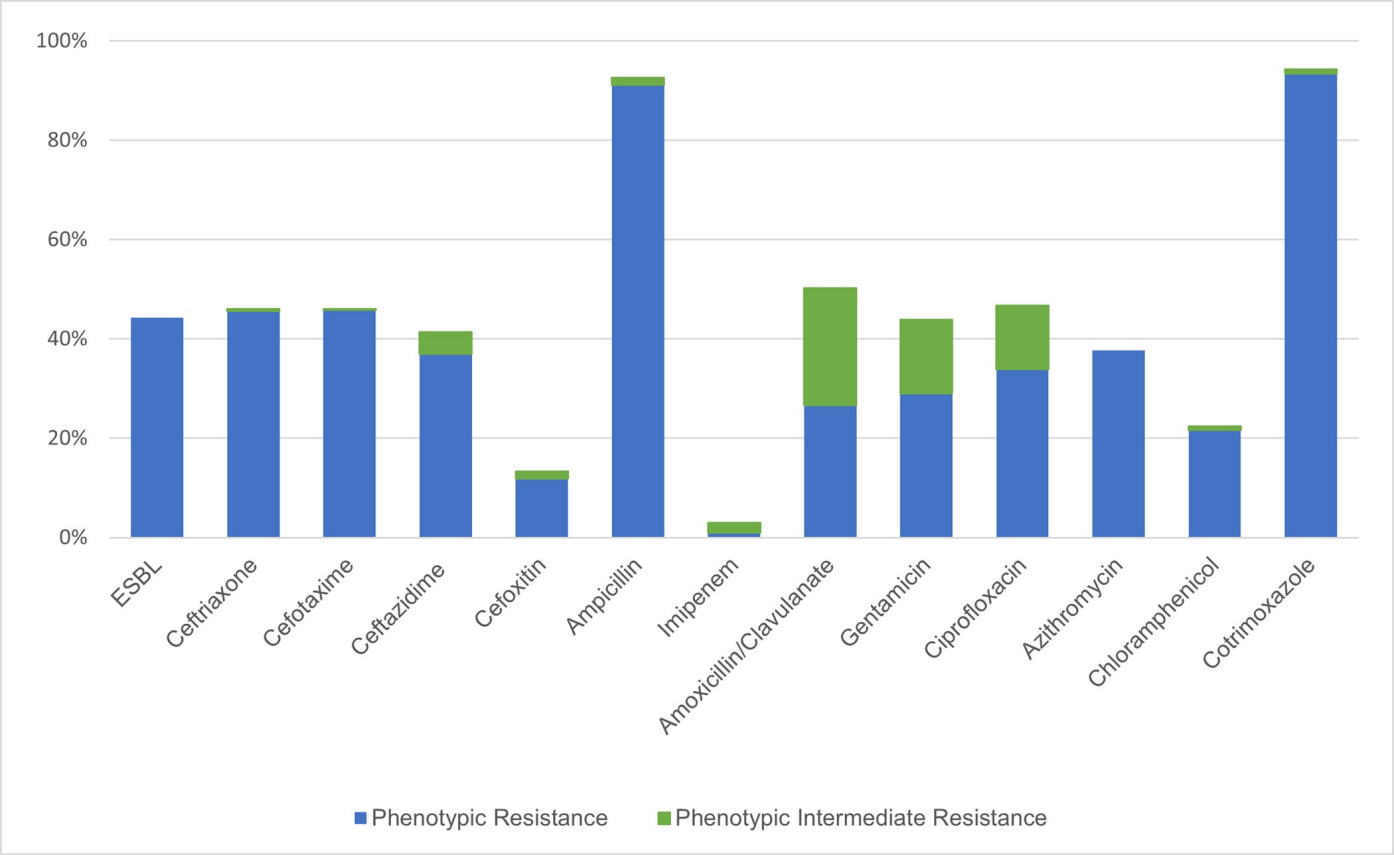
Prevalence of children with *E*. *coli* isolates at hospital discharge in Kenya resistant to selected antibiotics (N = 406). Resistant isolates are determined by the sizes of clearings around antibiotics measured using disc diffusion antimicrobial susceptibility testing and classified as “resistant” or “intermediate” by CLSI guidelines.

Of the 406 children included in this study, 94 had two morphologically distinct *E*. *coli* identified, and another 19 had three distinct morphologies tested, totaling 538 unique isolates. All 406 children harbored *E*. *coli* with reduced susceptibility to at least one of the tested antibiotics isolated from a fecal sample. Almost all (92.6%) children had *E*. *coli* isolated that lacked phenotypic susceptibility to ampicillin (95% Confidence Interval [CI]: 89.6%– 95.0%), while 43.8% (95% CI: 39% - 48.8%) of children harbored isolates resistant to gentamicin (Fig 2). Lack of susceptibility to ceftriaxone was common (46.1% [95% CI: 41.1% - 51.0%]) as was presence of ESBL (44.3% [95% CI: 39.4% - 49.3%]). Non-susceptibility to cephalosporins other than ceftriaxone was also commonly identified, with 46.1% (95% CI: 41.1% - 51.0%) of *E*. *coli* isolated from children had non-susceptibility to cefotaxime, and 41.4% (95% CI: 36.5% - 46.3%) to ceftazidime. There was relatively low resistance to cefoxitin identified (13.3% [95% CI: 10.2% - 17%]). About half of the children (50.6% (95% CI: 45.6% - 55.6%) had an *E*. *coli* isolate with reduced susceptibility to amoxicillin/clavulanic acid. Children also harbored *E*. *coli* with phenotypic non-susceptibility to less commonly used antibiotics including ciprofloxacin (46.8% [95% CI: 41.9% - 51.8%], azithromycin (37.7% [95% CI: 33% - 42.6%]), and chloramphenicol (22.7% [95% CI: 18.7%– 27.0%]). Only 3% (95% CI: 1.5% - 5.1%) of children were colonized with *E*. *coli* resistant to imipenem.

### Risk factors for ESBL producing *E*. *coli* in kenyan children at hospital discharge

Receiving any antibiotic while hospitalized was positively associated with the presence of ESBL producing *E*. *coli* (adjusted prevalence ratio [aPR] = 2.23; 95% CI: 1.29–3.83, Table 2). Specifically, receiving ceftriaxone while hospitalized was positively associated with the presence of ESBL producing *E*. *coli* (aPR = 3.01; 95% CI: 1.78–5.09) compared to not receiving any antibiotics. Prior hospitalization in the previous year was also positively associated with ESBL producing *E*. *coli* (aPR 1.32 [1.07–1.69]) as were hospital stays of at least 4 days (aPR = 1.34; 95% CI 1.07–1.69). A diagnosis of gastroenteritis (aPR = 1.42; 95% CI 1.08–1.89) was associated with ESBL-producing *E*. *coli* being isolated from fecal samples. Being diagnosed with meningitis was also positively associated with ESBL-producing bacteria (crude prevalence ratio [cPR] = 1.26; 95% CI 1.01–1.56), as was a diagnosis of malaria (cPR = 1.26; 1.01–1.56). However, these associations were not seen in adjusted models (aPR 0.90; 0.68–1.19 and 1.20; 95% CI 0.94–1.53, respectively). Other hospital diagnoses, enrolling hospital, nor whole stool versus rectal swab collection (S1 Table) were associated with higher presence of ESBL producing *E*. *coli*. The practice of open defecation was associated with ESBL-producing *E*. *coli* (aPR = 2.02; 95% CI: 1.39–2.94), as was the sharing of sanitation facilities with other households (aPR = 1.49; 95% CI: 1.17–1.89). Living in crowded housing and availability of improved water source were also not associated with ESBL producing *E*. *coli*. Female sex was the only host characteristic with increased likelihood of isolating ESBL-producing *E*. *coli* (aPR = 1.42; 95% CI: 1.15–1.76).

**Table 2:** Risk Factors for ESBL Producing *E*. *coli* from Fecal Samples.

	ESBL + (N = 177)		ESBL - (N = 229)		Prevalence Ratios (95% CI)	p-value	Adjusted Prevalence Ratios (95% CI)^i^	p-value
	N	(%)^ii^	N	(%)^ii^				
Facility Kisii Homa Bay	100 77	(56.1%) (43.9%)	142 87	(62.4%) (37.6%)	REF. 1.13 (0.91 – 1.42)	0.26	REF. 0.87 (0.69 – 1.11)	0.26
**Child Characteristics**								
Sex Male Female	98 79	(55.4%) (44.6%)	143 86	(62.5%) (37.6%)	REF. 1.18 (0.94 – 1.47)	0.15	REF. 1.42 (1.15 – 1.76)	0.00***
Age (months) 24 and over 12 – 23 6 – 11 1 – 5	76 49 30 22	(42.9%) (27.7%) (17.0%) (12.4%)	84 73 50 22	(36.7%) (31.9%) (21.8%) (9.6%)	REF. 0.85 (0.64 – 1.11) 0.79 (0.57 – 1.09) 1.05 (0.75 – 1.48)	0.23 0.16 0.77	REF. 1.01 (0.77 – 1.33) 1.24 (0.92 – 1.68) 1.23 (0.75 – 1.81)	0.92 0.16 0.49
Breastfeeding^iii^ Exclusively Breastfed Partially Breastfed Never Breastfed	87 79 1	(52.1%) (47.3%) (0.6%)	98 116 4	(45.0%) (53.2%) (1.8%)	REF. 0.45 (0.07 – 2.74) 0.91 (0.58 – 1.44)	0.39 0.69	REF. 1.21 (0.74 – 1.98) 0.64 (0.13 – 3.04)	0.44 0.58
HIV^iv^ HIV Uninfected HIV Uninfected, Exposed HIV Infected	150 15 5	(88.2%) (8.8%) (2.9%)	19028 3	(86.0%) (12.7%) (1.4%)	REF. 0.79 (0.52 – 1.21) 1.42 (0.82 – 2.45)	0.28 0.22	0.70 (0.41 – 1.21) 1.45 (0.91 – 2.31)	0.20 0.12
Nutritional Characteristics Neither stunted nor wasted Wasted, not Stunted Stunted, not Wasted Stunted and Wasted	119 12 40 6	(67.2%) (6.8%) (22.6%) (3.4%)	153 22 49 5	(66.8%) (9.6%) (21.4%) (2.2%)	REF. 0.81 (0.50 – 1.30) 1.03 (0.79 – 1.34) 1.25 (0.71 – 2.18)	0.38 0.84 0.44	0.75 (0.46 – 1.21) 1.03 (0.76 – 1.40) 0.81 (0.31 – 2.11)	0.24 0.84 0.67
**Hospitalization Information**								
Referred from Another Health Facility No Yes	125 52	(70.6%) (29.4%)	175 54	(76.4%) (23.6%)	REF. 1.18 (0.93 – 1.49)	0.18	REF. 0.77 (0.53 – 1.12)	0.17
Hospitalizations in the Prior Year^v^ No Yes	132 49	(72.9%) (27.1%)	189 34	(84.8%) (15.3%)	REF. 1.41 (1.13 – 1.77)	0.00***	REF. 1.32 (1.07 – 1.64)	0.01*
Length of Hospital Stay (in days)^vi^ < 4 ≥ 4	78 96	(44.8%) (55.2%)	135 93	(59.2%) (40.8%)	REF 1.40 (1.12 – 1.75)	0.03***	REF. 1.34 (1.07 – 1.69)	0.01*
Received Antibiotic During Hospitalization No Yes	12 165	(6.8%) (93.2%)	40 189	(17.5%) (82.5%)	REF. 2.02 (1.21 – 3.36)	0.01***	REF. 2.23 (1.29 – 3.83)	0.00***
Antibiotic Received During Hospitalization^vii^ Penicillins Ceftriaxone Gentamicin	92 95 81	(88.5%) (88.8%) (87.1%)	155 36 138	(79.5%) (47.4%) (77.5%)	1.61 (0.96 – 2.72) 3.14 (1.90 – 5.23) 1.69 (0.95 – 2.71)	0.07 0.00*** 0.08	1.73 (0.96 – 3.12) 3.01 (1.78 – 5.09) 1.68 (0.93 – 3.06)	0.07 0.00*** 0.09
Admitting Diagnosis^viii^ Anemia Gastroenteritis/Diarrhea Malaria Meningitis Pneumonia/LRTI	39 31 94 25 55	(22.0%) (17.5%) (53.1%) (12.1%) (31.1%)	44 52 98 16 91	(19.2%) (22.7%) (42.8%) (7.0%) (39.7%)	1.07 (0.82 – 1.39) 0.84 (0.62 – 1.12) 1.26 (1.01 – 1.56) 1.43 (1.09 – 1.87) 0.82 (0.64 – 1.04)	0.62 0.24 0.04* 0.01* 0.10	1.05 (0.81 – 1.34) 1.42 (1.08 – 1.89) 1.20 (0.94 – 1.53) 0.90 (0.68 – 1.19) 0.91 (0.70 – 1.18)	0.72 0.01* 0.15 0.45 0.46
**Household Information**								
Crowding No Yes	97 80	(54.8%) (45.2%)	117 112	(51.1%) (48.9%)	REF. 0.92 (0.74 – 1.15)	0.46	REF. 1.02 (0.83 – 1.25)	0.88
Livestock Ownership No Yes	59 118	(33.3%) (66.7%)	61 168	(26.6%) (73.4%)	REF. 0.84 (0.67 – 1.05)	0.13	REF. 0.93 (0.52 – 1.16)	0.52
Improved Water Source No Yes	28 149	(15.8%) (84.2%)	37 192	(16.2%) (83.8%)	REF. 1.01 (0.75 – 1.38)	0.93	REF. 1.07 (0.82 – 1.39)	0.62
Treated Drinking Water^ix^ No Yes	89 91	(49.4%) (50.6%)	112 109	(50.7%) (49.3%)	REF. 1.02 (0.83 – 1.28)	0.81	REF. 0.91 (0.72 – 1.16)	0.45
Toilet^x^ Private, for Household Only Shared with ≥1 Other Household Open Defecation	73 93 14	(40.6%) (51.7%) (7.8%)	109 107 6	(49.1%) (48.2%) (2.7%)	REF. 1.16 (0.92 – 1.46) 1.75 (1.24 – 2.35)	0.21 0.00***	REF. 1.49 (1.17 – 1.89) 2.02 (1.39 – 2.94)	0.00*** 0.00***

^i^Adjusted for a priori determined potential confounders (facility, age, gender) in addition to variables deemed associated in univariate models at the p ≤.05 level (length of hospital stay, in-hospital use of cephalosporins, whether any hospitalizations occurred within the year preceding enrollment, toilet, and diagnosis of meningitis or malaria at admission). The multivariable model did not include whether or not an antibiotic was received in the hospital due to potential collinearity with cephalosporin use.

^ii^Column Percentages shown

^iii^Current breastfeeding for children ≤6 months and breastfeeding practiced when children were under 6 months; n = 21 unknown

^iv^Uninfected, Exposure Status unknown (n = 11), Exposed, infection status unknown (n = 4); Column percentages of children with exposure and infection status known (n = 390)

^v^Of the 404 with previous hospitalizations, if any, known

^vi^Among those with both admission and discharge dates available (n = 401)

^vii^Reference group are those who did not receive an antibiotic (n = 52), which make up the denominator of the column percentage shown (12 of those who did not receive an antibiotic had ESBL-producing *E*. *coli* isolated, while 38 did not have ESBL-producing isolates). Antibiotics given in hospital were not mutually exclusive. Other antibiotics given: azithromycin (n = 4), cefuroxime (n = 5), trimethoprim-cotrimoxazole (n = 10), chloramphenicol (n = 16), ciprofloxacin (n = 1), clarithromycin (n = 5), erythromycin (n = 1), tetracycline (n = 1), metronidazole (n = 18). Of the antibiotics tested: 247 were given a penicillin class antibiotic(s). Of these, a total of 155 did not have ESBL-producing *E*. *coli* isolated, while 92 given a penicillin drug had ESBL-producing *E*. *coli* isolated. 131 were given ceftriaxone; 36 did not have ESBL-producing bacteria isolated, while 95 did. 219 children were given gentamicin, 63% of whom did not have ESBL-producing bacteria. 53 children received antibiotics not belonging to the prior three classes, 25 had ESBL-producing E. coli isolated.

^viii^Not mutually exclusive. The reference group for each diagnosis from the time of admission is not having the corresponding diagnosis. Other diagnoses at admission include: asthma (n = 7), HIV (n = 2), poisoning/herbal toxicity (n = 4), UTI (n = 2)

^ix^Treated water included those who did not report use of bottled water and reported filters, boiling, or chlorinating to treat drinking water. The comparison group was those who did not treat drinking water and did not use bottled water (n = 401)

^x^Of those who answered (n = 402)

***Significant at an alpha of 0.01 *Significant at an alpha of 0.05

When adjusting for variables chosen *a priori* (sex, age, and facility) female sex was no longer associated with carriage of ESBL *E*. *coli* (aPR = 1.20; 0.97–1.49) nor was sharing a toilet with at least 1 other household (aPR 1.16; 0.92–1.47) (S2 Table). In this secondary analysis, meningitis was associated with carriage of ESBL-producing *E*. *coli* in the adjusted model (aPR = 1.43; 1.09–1.87) while other diagnoses did not demonstrate any association. As in the primary multivariate analyses, hospitalization in the prior year (aPR = 1.39; 1.11–1.76), length of hospital stay of 4 days or greater (aPR 1.39; 1.11–1.74), receiving an antibiotic (aPR = 1.93; 1.19–3.12), receiving ceftriaxone (aPR = 2.66; 1.66–4.26), and open defecation (aPR = 1.66; 1.14–2.41) were associated with higher carriage of ESBL-producing *E*. *coli*.

## Discussion

In this study of children recently discharged from two hospitals in Western Kenya, a high burden of AMR was identified. AMR is associated with mortality, longer hospital stays, and high treatment costs [32]. Factors related to hospitalization, including recent antibiotic use, were strongly associated with ESBL-producing *E*. *coli*. Antibiotic use is common in this population of severely ill hospitalized children and complicated by a lack of available diagnostic tests to rule out non-bacterial causes. The high rates of AMR carriage may be particularly problematic during the post-discharge period, when children are at high risk of subsequent illness.

Most children in this study received antibiotics recommended in national and international guidelines for the management of common childhood infections, including beta-lactam and/or aminoglycoside antibiotics [4,33,34]. Recent receipt of an antibiotic more than doubled the likelihood of having ESBL-producing *E*. *coli* isolated, suggesting that the frequent use of these drugs in hospitals leads to significant drug pressure driving AMR, as has been described previously [34]. Lack of susceptibility to ceftriaxone, a commonly used second-line antibiotic for many infections, was present in over 40% of children with *E*. *coli* isolates. The presence of ESBL was similarly common. These resistance rates are similar to those reported among hospitalized children in other East African settings [19]. The high rate of ESBL documented here is concerning given how critical beta-lactam antibiotics are to treatment of common illnesses. In addition, the presence of these beta-lactamases are also associated with resistance to aminoglycosides, another commonly antimicrobial class used in many settings [35]. Being hospitalized within the prior year was also associated with carrying ESBL-producing *E*. *coli*. Children with prior hospitalization may have been more likely to have received antibiotics or to have been exposed to a community in which antibiotic use drove selection pressure.

We found 44.3% of children to harbor ESBL-producing *E*. *coli*, as determined by examining 3^rd^ generation cephalosporins with and without the beta-lactamase inhibitor clavulanic acid. By contrast, resistance to individual 3^rd^ generation cephalosporins tested ranged from 41.4% (ceftazidime) to 46.1% (ceftriaxone and cefotaxime). Although we report phenotypic resistance patterns for individual antibiotics, clinicians may avoid treating with any 3^rd^ generation cephalosporin if there is resistance reported to another drug in the same class as the presence of broader resistance by ESBL-producing bacteria may result in the avoidance of most beta-lactams.

Nearly all (94%) of *E*. *coli* isolates were resistant to trimethoprim-sulfamethoxazole, despite only 2% of children having received trimethoprim-sulfamethoxazole during their hospital stay. Other studies in Sub-Saharan Africa have reported similarly high rates of trimethoprim-sulfamethoxazole resistance among children [18]. The high rate of resistance observed, despite the relatively low in-hospital use, likely reflects the widespread use of daily trimethoprim-sulfamethoxazole for persons living with HIV and for HIV-exposed children in many communities in the region [36]. Individual exposure may be less important in driving trimethoprim-sulfamethoxazole resistance than exposure to widespread resistance in the community, as has been observed elsewhere [37].

We found female sex to be associated with ESBL-producing *E*. *coli* as has been previously reported [38]. However, this relationship has not been consistently observed in other studies in SSA [19]. In low resource settings, female children may be more likely to experience delays in care seeking [39], which may lead to more severe disease by the time of presentation. Additionally, female children may be more likely to participate in duties for the household, potentially resulting in greater exposure to contaminated environments. This may result in the need for extended antimicrobial therapies and longer exposure to the hospital environment.

Child age, living in a crowded household environment, use of an improved water source, access to treated water, and livestock ownership were not associated with AMR in this analysis, although these findings differ from those reported in other studies in SSA [3,22,40]. Sanitation and hygiene in the forms of open defecation or sharing a toilet with other households were related to carriage of ESBL-producing bacteria, suggesting that not all ESBL was acquired in hospital [3]. The fact that children recently discharged from hospital harbor high levels of bacteria resistant to multiple classes of antibiotics used for the treatment of common infectious syndromes is a major public health concern due to potential implications for the broader population given sharing of AMR organisms or genetic elements to others in the household and community. Such sharing of AMR has been documented [41], and is likely most pronounced in crowded household environments with limited sanitation and hygiene facilities common in some rural SSA settings [21].

Although hospital-based antibiotic stewardship programs are a cornerstone of AMR prevention in many high-resource settings, in low-resource settings such programs may face additional challenges to implementation due to a reliance on syndromic diagnosis in the absence of diagnostic confirmatory testing, the limited availability of resistance testing and monitoring, difficulties in access to care and limited alternative antibiotic treatment options [4,42]. In SSA antibiotics are available outside of hospital and clinical settings which may reduce the effectiveness of hospital-based antibiotic stewardship programs in the region [5]. Some success has been seen with antibiotic stewardship programs in South Africa without increasing mortality caused by infectious diseases, which may be an adverse consequence of limiting antibiotic use in these settings [43,44].

There were several strengths to this analysis. Few studies have examined carriage of AMR bacteria in children at hospital discharge. In addition, most studies focus on pathogenic bacteria although AMR transmission from commensal flora may be important to community spread [21]. Additionally, AMR may be part of the high rate of rehospitalization and death in the post-discharge period. The study also was conducted in a rural setting which adds to available data from major African cities where other studies have been conducted previously [17,19,22]. However, this study does have several limitations. By limiting our study population to children who survived hospitalization, we may have preferentially selected children less likely to be harboring resistance. Due to this study’s cross-section design we are unable to differentiate AMR that was acquired prior to a child’s hospitalization from that acquired in-hospital nor were we able to discern risk-factors that may be associated with carriage of ESBL-producing *E*. *coli* prior to discharge. However, hospitalizations in children under the age of 5 years are common in western Kenya, suggesting our study population may be relatively generalizable to a sizeable proportion of children in the region [45–48]. Future studies should include assessments of AMR at admission, discharge, and into the post-discharge period to further discern when resistant *E*. *coli* is acquired. We evaluated a relatively small number of isolates, which means we may have missed AMR present in non-sampled isolates from these children.

## Conclusions

The prevalence of AMR in *E*. *coli* from children who have been hospitalized and subsequently discharged from hospital in Western Kenya is high. AMR is increasing globally and the public health relevance of these findings is likely significant, both for individual children and for communities. Health care exposure appears to be a major driver of AMR and interventions to prevent and reduce AMR transmission and acquisition in the health care setting are urgently needed. Interventions are urgently needed that include the prevention of illnesses that lead to children being hospitalized and attention to alternative antimicrobial therapies for children who experience AMR-related clinical failure in LMIC settings.

## Supporting information

S1 Table Association of swabs or whole stool and the identification of ESBL-Producing *E*. *coli*. (DOCX)

S2 Table Risk Factors for ESBL Producing *E*. *coli* from Fecal Samples with Multivariate Models adjusting solely for *a priori* defined variables. (DOCX)

S3 Table Comparison of characteristics of children selected for the AMR study compared to the other children in the parent study eligible for sampling. (DOCX)

S4 Table Comparison of characteristics of children selected for the AMR study with E. coli compared to children without *E*. *coli* isolated from fecal samples. (DOCX)
