# Supplementary material for: Antimicrobial resistance including Extended Spectrum Beta Lactamases (ESBL) among E. coli isolated from kenyan children at hospital discharge
Source: PLoS Negl Trop Dis. 2022 Mar 31;16(3):e0010283. doi: 10.1371/journal.pntd.0010283 (PMC9015121; doi:10.1371/journal.pntd.0010283)
Supplement: S1 Table — (DOCX) [file pntd.0010283.s001.docx]

**Appendix I.** Association of swabs or whole stool and the identification of ESBL-Producing *E. coli*.

|  | **ESBL Producing** | | **ESBL Negative** | | **Prevalence Ratio (95% CI)** | **p-value** |
| --- | --- | --- | --- | --- | --- | --- |
|  | **N** | **(%)** | **N** | **(%)** |  |  |
| **ESBL**  Whole Stool  Rectal Swab | 21  156 | (11.9%)  (88.1%) | 48  181 | (21.0%)  (79.0%) | REF.  1.27 (0.87 – 1.86) | 0.22 |
