# Supplementary material for: Antimicrobial resistance including Extended Spectrum Beta Lactamases (ESBL) among E. coli isolated from kenyan children at hospital discharge
Source: PLoS Negl Trop Dis. 2022 Mar 31;16(3):e0010283. doi: 10.1371/journal.pntd.0010283 (PMC9015121; doi:10.1371/journal.pntd.0010283)
Supplement: S2 Table — (DOCX) [file pntd.0010283.s002.docx]

**Appendix II.** Risk Factors for ESBL Producing *E. coli* from Fecal Samples with Multivariate Models adjusting solely for *a priori* defined variables

|  | **ESBL +**  **(N = 177)** | | **ESBL -**  **(N = 229)** | | **Prevalence Ratio (95% CI)** | **p-value** | **Adjusted Prevalence Ratio (95% CI)^[[1]](#endnote-1)^** | **p-value** |
| --- | --- | --- | --- | --- | --- | --- | --- | --- |
|  | **N** | **(%)^[[2]](#endnote-2)^** | **N** | **(%)^i^** |  |  |  |  |
| Facility  Kisii  Homa Bay | 100  77 | (56.1%)  (43.9%) | 142  87 | (62.4%)  (37.6%) | REF.  1.13 (0.91 – 1.42) | 0.26 | REF.  1.14 (0.91 – 1.41) | 0.25 |
| **Child Characteristics** |  |  |  |  |  |  |  |  |
| Sex  Male  Female | 98  79 | (55.4%)  (44.6%) | 143  86 | (62.5%)  (37.6%) | REF.  1.18 (0.94 – 1.47) | 0.15 | REF.  1.20 (0.97 – 1.49) | 0.09 |
| Age (months)  24 and over  12 – 23  6 – 11  1 – 5 | 76  49  30  22 | (42.9%)  (27.7%)  (17.0%)  (12.4%) | 84  73  50  22 | (36.7%)  (31.9%)  (21.8%)  (9.6%) | REF.  0.85 (0.64 – 1.11)  0.79 (0.57 – 1.09)  1.05 (0.75 – 1.48) | 0.23  0.16  0.77 | REF.  1.03 (0.67 – 1.14)  1.08 (0.62 – 1.88)  1.50 (0.80 – 2.81) | 0.32  0.39  0.52 |
| Breastfeeding^[[3]](#endnote-3)^  Exclusively Breastfed  Partially Breastfed  Never Breastfed | 87  79  1 | (52.1%)  (47.3%)  (0.6%) | 98  116  4 | (45.0%)  (53.2%)  (1.8%) | REF.  0.45 (0.07 – 2.74)  0.91 (0.58 – 1.44) | 0.39  0.69 | REF.  0.90 (0.57 – 1.41)  0.43 (0.07 – 2.61) | 0.64  0.36 |
| HIV^[[4]](#endnote-4)^  HIV Uninfected  HIV Uninfected, Exposed  HIV Infected | 150  15  5 | (88.2%)  (8.8%)  (2.9%) | 19028  3 | (86.0%)  (12.7%)  (1.4%) | REF.  0.79 (0.52 – 1.21)  1.42 (0.82 – 2.45) | 0.28  0.22 | 0.74 (0.48 – 1.13)  1.23 (0.71 – 2.13) | 0.16  0.47 |
| Nutritional Characteristics  Neither stunted nor wasted  Wasted, not Stunted  Stunted, not Wasted  Stunted and Wasted | 119  12  40  6 | (67.2%)  (6.8%)  (22.6%)  (3.4%) | 153  22  49  5 | (66.8%)  (9.6%)  (21.4%)  (2.2%) | REF.  0.81 (0.50 – 1.30)  1.03 (0.79 – 1.34)  1.25 (0.71 – 2.18) | 0.38  0.84  0.44 | 0.78 (0.48 – 1.26)  1.02 (0.78 – 1.33)  1.22 (0.72 – 2.04) | 0.32  0.88  0.46 |
| **Hospitalization Information** |  |  |  |  |  |  |  |  |
| Referred from Another Health Facility  No  Yes | 125  52 | (70.6%)  (29.4%) | 175  54 | (76.4%)  (23.6%) | REF.  1.18 (0.93 – 1.49) | 0.18 | REF.  1.30 (0.95 – 1.79) | 0.10 |
| Hospitalization within the Prior Year^[[5]](#endnote-5)^  No  Yes | 132  49 | (72.9%)  (27.1%) | 189  34 | (84.8%)  (15.3%) | REF.  1.41 (1.13 – 1.77) | 0.00*** | REF.  1.39 (1.11 – 1.76) | 0.01*** |
| Length of Hospital Stay (in days)  < 4  ≥ 4 | 78  96 | (44.8%)  (55.2%) | 135  93 | (59.2%)  (40.8%) | REF  1.40 (1.12 – 1.75) | 0.03*** | REF.  1.39 (1.11 – 1.74) | 0.00*** |
| Received Antibiotic During Hospitalization  No  Yes | 12  165 | (6.8%)  (93.2%) | 40  189 | (17.5%)  (82.5%) | REF.  2.02 (1.21 – 3.36) | 0.01*** | REF.  1.93 (1.19 – 3.12) | 0.01*** |
| Antibiotic Received During Hospitalization^[[6]](#endnote-6)^  Penicillins  Ceftriaxone  Gentamicin | 92  95  81 | (88.5%)  (88.8%)  (87.1%) | 155  36  138 | (79.5%)  (47.4%)  (77.5%) | 1.61 (0.96 – 2.72)  3.14 (1.90 – 5.23)  1.69 (0.95 – 2.71) | 0.07  0.00***  0.08 | 1.48 (0.87 – 2.52)  2.66 (1.66 – 4.26)  1.40 (0.81 – 2.44) | 0.15  0.00***  0.23 |
| Admitting Diagnosis^[[7]](#endnote-7)^  Anemia  Gastroenteritis/Diarrhea  Malaria  Meningitis  Pneumonia/LRTI | 39  31  94  25  55 | (22.0%)  (17.5%)  (53.1%)  (12.1%)  (31.1%) | 44  52  98  16  91 | (19.2%)  (22.7%)  (42.8%)  (7.0%)  (39.7%) | 1.07 (0.82 – 1.39)  0.84 (0.62 – 1.12)  1.01 (1.01 – 1.56)  1.43 (1.09 – 1.87)  0.82 (0.64 – 1.04) | 0.62  0.24  0.04*  0.01*  0.10 | 1.04 (0.79 – 1.36)  0.86 (0.63 – 1.16)  1.23 (0.99 – 1.54)  1.43 (1.09 – 1.87)  0.85 (0.66 – 1.09) | 0.78  0.31  0.07  0.01***  0.20 |
| **Household Information** |  |  |  |  |  |  |  |  |
| Crowding  No  Yes | 97  80 | (54.8%)  (45.2%) | 117  112 | (51.1%)  (48.9%) | REF.  0.92 (0.74 – 1.15) | 0.46 | REF.  0.89 (0.71 – 1.11) | 0.30 |
| Livestock Ownership  No  Yes | 59  118 | (33.3%)  (66.7%) | 61  168 | (26.6%)  (73.4%) | REF.  0.84 (0.67 – 1.05) | 0.13 | REF.  0.83 (0.66 – 1.05) | 0.11 |
| Improved Water Source  No  Yes | 28  149 | (15.8%)  (84.2%) | 37  192 | (16.2%)  (83.8%) | REF.  1.01 (0.75 – 1.38) | 0.93 | REF.  0.96 (0.72 – 1.28) | 0.78 |
| Treated Drinking Water  No  Yes | 89  91 | (49.4%)  (50.6%) | 112  109 | (50.7%)  (49.3%) | REF.  1.02 (0.83 – 1.28) | 0.81 | REF.  0.97 (0.77 – 1.23) | 0.82 |
| Toilet^[[8]](#endnote-8)^  Private, for Household Only  Shared with ≥1 Other Household  Open Defecation | 71  91 | (43.8%)  (56.2%) | 111  109 | (50.5%)  (49.6%) | REF.  1.16 (0.92 – 1.47)  1.66 (1.14 - .2.41) | 0.21  0.01*** | REF.  1.16 (0.92 – 1.47)  1.66 (1.14 – 2.41) | 0.21  0.01*** |

1. Adjusted for a priori determined potential confounders (facility, age, sex). [↑](#endnote-ref-1)
2. Column Percentages shown [↑](#endnote-ref-2)
3. Current breastfeeding for children ≤6 months and breastfeeding practiced when children were under 6 months; n = 21 unknown [↑](#endnote-ref-3)
4. Uninfected, Exposure Status unknown (n = 11), Exposed, infection status unknown (n = 4); Column percentages of children with exposure and infection status known (n = 390) [↑](#endnote-ref-4)
5. Of those with hospitalizations in the prior year known (n = 404) [↑](#endnote-ref-5)
6. Reference group are those who did not receive an antibiotic (n = 52), which make up the denominator of the column percentage shown (12 of those who did not receive an antibiotic had ESBL-producing *E. coli* isolated, while 38 did not have ESBL-producing isolates). Antibiotics given in hospital were not mutually exclusive. Other antibiotics given: azithromycin (n = 4), cefuroxime (n = 5), trimethoprim-cotrimoxazole (n = 10), chloramphenicol (n = 16), ciprofloxacin (n = 1), clarithromycin (n = 5), erythromycin (n = 1), tetracycline (n = 1), metronidazole (n = 18). Of the antibiotics tested: 247 were given a penicillin class antibiotic(s). Of these, a total of 155 did not have ESBL-producing *E. coli* isolated, while 92 given a penicillin drug had ESBL-producing *E. coli* isolated. 131 were given ceftriaxone; 36 did not have ESBL-producing bacteria isolated, while 95 did. 219 children were given gentamicin, 63% of whom did not have ESBL-producing bacteria. 53 children received antibiotics not belonging to the prior three classes, 25 had ESBL-producing E. coli isolated. [↑](#endnote-ref-6)
7. Not mutually exclusive. The reference group for each diagnosis is not having the corresponding diagnosis. Other diagnoses at admission include: asthma (n = 7), HIV (n = 2), poisoning/herbal toxicity (n = 4), UTI (n = 2) [↑](#endnote-ref-7)
8. Of those who do not practice open defecation (n = 381)

   ***Significant at an alpha of 0.01 *Significant at an alpha of 0.05 [↑](#endnote-ref-8)
