# Supplementary material for: Antimicrobial resistance including Extended Spectrum Beta Lactamases (ESBL) among E. coli isolated from kenyan children at hospital discharge
Source: PLoS Negl Trop Dis. 2022 Mar 31;16(3):e0010283. doi: 10.1371/journal.pntd.0010283 (PMC9015121; doi:10.1371/journal.pntd.0010283)
Supplement: S3 Table — (DOCX) [file pntd.0010283.s003.docx]

**Appendix III.** Comparison of characteristics of children selected for the AMR study compared to the other children in the parent study eligible for sampling

|  | **Selected for AST Testing (n = 455)** | | **Not Selected for AST Testing (n = 890)** | | **p-value** |
| --- | --- | --- | --- | --- | --- |
|  | **n** | **(%)** | **n** | **(%)** |  |
| **Participant Characteristics** |  |  |  |  |  |
| Sex |  |  |  |  |  |
| Male  Female | 271  184 | (59.6%)  (40.4%) | 526  364 | (59.1%)  (40.9%) | 0.87 |
| Age (months) |  |  |  |  |  |
| 24 and over  12 – 23  6 – 11  1 – 5 | 170  140  90  55 | (37.4%)  (30.8%)  (19.8%)  (12.1%) | 332  253  176  129 | (37.3%)  (28.4%)  (19.8%)  (14.5%) | 0.61 |
| Breastfeeding^[[1]](#endnote-1)^  Exclusively Breastfed  Partially Breastfed  Never Breastfed | 217  210  5 | (50.2%)  (48.6%) (1.2%) | 443  421  19 | (50.2%)  (47.7%) (2.2%) | 0.42 |
| HIV Status^[[2]](#endnote-2)^ |  |  |  |  |  |
| HIV Uninfected  HIV Uninfected, Exposed  HIV Infected | 381  47  10 | (87.0%) (10.7%)  (2.3%) | 763  88  8 | (88.8%)  (10.2%)  (0.9%) | 0.14 |
| Nutritional Characteristics^[[3]](#endnote-3)^  Neither Stunted nor Wasted  Wasted, not Stunted  Stunted, not Wasted  Stunted and Wasted | 304  39  101  11 | (66.8%)  (8.6%)  (22.2%)  (2.4%) | 668  62  141  19 | (75.1%)  (8.6%)  (22.2%)  (2.4%) | 0.01* |
| **Hospitalization Information** |  |  |  |  |  |
| Length of Hospital Stay (in days)^[[4]](#endnote-4),^^[[5]](#endnote-5)^ | 3 | (2 – 5) | 3 | (2 – 5) | 0.95 |
| Received Antibiotic during Hospitalization | 401 | (88.3%) | 801 | (90.0%) | 0.29 |
| Antibiotic Received during Hospitalization^[[6]](#endnote-6)^  Penicillins  Ceftriaxone  Gentamicin  Other | 278  150  250  66 | (69.3%)  (37.4%)  (62.3%) (16.5%) | 551  362  482  119 | (68.8%)  (45.2%)  (60.2%)  (14.9%) | 0.85  0.01*  0.47  0.47 |
| Admitting Diagnosis^[[7]](#endnote-7)^  Anemia  Gastroenteritis/Diarrhea  Malaria  Meningitis  Pneumonia/LRTI  Sickle Cell  Suspected Sepsis  Tuberculosis  Other | 93  94  239  49  164  49  17  11  15 | (20.4%)  (20.7%)  (52.5%)  (10.8%)  (36.0%)  (10.8%)  (3.7%)  (2.4%)  (3.3%) | 145  163  452  115  356  71  55  28  55 | (16.3%)  (18.3%)  (50.8%)  (12.9%)  (40.0%)  (8.0%)  (6.2%)  (3.2%)  (6.2%) | 0.06  0.30  0.55  0.25  0.16  0.09  0.06  0.45  0.02* |
| Prescribed Antibiotic at Discharge | 275 | (60.4%) | 558 | (62.7%) | 0.42 |
| **Household Information** |  |  |  |  |  |
| Crowding (>2 people/room) | 211 | (46.4%) | 407 | (45.7%) | 0.82 |
| Livestock Ownership | 316 | (69.5%) | 587 | (66.0%) | 0.28 |
| Improved Water Source^[[8]](#endnote-8)^ (31) | 379 | (83.3%) | 728 | (81.8%) | 0.50 |
| Shared Toilet^[[9]](#endnote-9)^ | 225 | (52.5%) | 389 | (45.5%) | 0.02* |
| Toilet Type  Flushing  Pit Latrine  Open Defecation | 37  398  20 | (8.1%)  (87.5%)  (4.4%) | 83  774  32 | (9.3%)  (87.1%)  (3.6%) | 0.33 |

1. Of those with data available (n = 1,268) Current breastfeeding for children ≤6 months or breastfeeding practiced when children were under 6 months; n = 77 unknown [↑](#endnote-ref-1)
2. Uninfected, Exposure Status unknown (n = 41), Exposed, infection status unknown (n = 7); Column percentages of children with exposure and infection status known (n = 1,297) [↑](#endnote-ref-2)
3. Wasted is defined as WHZ < -2 or MUAC <11.5cm while Stunted is determined by HAZ <-2; MUAC is only taken into consideration in children 6 months or older [↑](#endnote-ref-3)
4. Median and interquartile range provided [↑](#endnote-ref-4)
5. Of those with admission and discharge dates both available (n = 1,336) [↑](#endnote-ref-5)
6. Not mutually exclusive. Total n = 1,202 (89.4%) received antibiotics, column percentages are of these children. Other antibiotics given: azithromycin (n = 23), co-amoxiclav (n = 1), cefuroxime (n = 16), trimethoprim-sulfamethoxazole (n = 22), chloramphenicol (n = 41), ciprofloxacin (n = 8), clarithromycin (n = 22), erythromycin (n = 7), tetracycline (n = 6), metronidazole (n = 60) [↑](#endnote-ref-6)
7. Not mutually exclusive. Other diagnoses at admission include: HIV (n = 5), urinary tract infection (n = 16), poisoning/herbal toxicity (n = 14), asthma (n = 34), upper respiratory tract infection (n = 91), fever of unknown origin (n = 2), and unknown (n = 70) [↑](#endnote-ref-7)
8. Those had drinking water included participants who did not report use of bottled water and responded using filters, boiling, or chlorinating drinking water [↑](#endnote-ref-8)
9. Shared Toilets are those used by more than 1 household and did not include open defecation (n = 52) and excluding those who did not answer (n = 9) [↑](#endnote-ref-9)
