# Supplementary material for: Antimicrobial resistance including Extended Spectrum Beta Lactamases (ESBL) among E. coli isolated from kenyan children at hospital discharge
Source: PLoS Negl Trop Dis. 2022 Mar 31;16(3):e0010283. doi: 10.1371/journal.pntd.0010283 (PMC9015121; doi:10.1371/journal.pntd.0010283)
Supplement: S4 Table — (DOCX) [file pntd.0010283.s004.docx]

**Appendix IV.** Comparison of characteristics of children selected for the AMR study with E. coli compared to children without *E. coli* isolated from fecal samples

|  | **AST Testing**  **n** | **(n = 406)**  **(%)** | **No AST Testing**  **n** | **(n = 42)**  **(%)** | **p-value** |
| --- | --- | --- | --- | --- | --- |
| **Participant Characteristics** |  |  |  |  |  |
| Sex |  |  |  |  |  |
| Male  Female | 241  165 | (59.4%)  (40.6%) | 25  17 | (59.5%)  (40.5%) | 0.98 |
| Age (months) |  |  |  |  |  |
| 24 and over  12 – 23  6 – 11  1 – 5 | 160  122  80  44 | (39.4%)  (30.1%)  (19.7%)  (10.8%) | 8  15  9  10 | (19.1%)  (35.7%)  (21.4%)  (23.8%) | 0.02* |
| Breastfeeding^[[1]](#endnote-1)^  Exclusively Breastfed  Partially Breastfed  Never Breastfed | 185  195  5 | (48.1%)  (50.7%) (1.3%) | 28  13  0 | (68.3%)  (31.7%) (0%) | 0.04 |
| HIV Status^[[2]](#endnote-2)^ |  |  |  |  |  |
| HIV Uninfected  HIV Uninfected, Exposed  HIV Infected | 340  43  8 | (87.0%) (11.0%)  (2.1%) | 35  4  1 | (87.5%)  (10.0%)  (2.5%) | 0.97 |
| Nutritional Characteristics^[[3]](#endnote-3)^  Neither Stunted nor Wasted  Wasted, not Stunted  Stunted, not Wasted  Stunted and Wasted | 273  34  88  11 | (67.2%)  (8.4%)  (21.7%)  (2.7%) | 30  3  9  0 | (67.0%)  (7.1%)  (21.4%)  (2.0%) | 0.03* |
| **Hospitalization Information** |  |  |  |  |  |
| Length of Hospital Stay (in days)^[[4]](#endnote-4)^^[[5]](#endnote-5)^ | 3 | (2 – 5) | 4 | (2 – 5) | 0.26 |
| Received Antibiotic during Hospitalization | 354 | (87.2%) | 41 | (97.6%) | 0.046* |
| Antibiotic Received during Hospitalization^[[6]](#endnote-6)^  Penicillins  Ceftriaxone  Gentamicin  Other | 247  133  219  53 | (69.8%)  (77.6%)  (61.9%) (15.0%) | 25  19  25  9 | (96.2%)  (95.0%)  (96.2%)  (90.0%) | 0.07  0.03*  0.05  0.75 |
| Admitting Diagnosis^[[7]](#endnote-7)^  Anemia  Gastroenteritis/Diarrhea  Malaria  Meningitis  Pneumonia/LRTI  Sickle Cell  Suspected Sepsis  Tuberculosis  URTI  Other | 83  83  192  41  146  43  13  11  28  15 | (20.4%)  (20.4%)  (47.3%)  (10.1%)  (36.0%)  (10.6%)  (3.2%)  (2.7%)  (6.9%)  (3.7%) | 8  9  21  8  14  6  4  0  3  8 | (19.1%)  (21.4%)  (50.0%)  (19.1%)  (33.3%)  (14.3%)  (9.5%)  (0%)  (7.1%)  (19.1%) | 0.83  0.88  0.74  0.08  0.74  0.47  0.04*  0.79  0.95  0.83 |
| Prescribed Antibiotic at Discharge | 242 | (59.8%) | 29 | (69.1%) | 0.23 |
| **Household Information** |  |  |  |  |  |
| Crowding (>2 people/room) | 192 | (47.3%) | 17 | (40.5%) | 0.40 |
| Livestock Ownership | 286 | (70.4%) | 26 | (61.9%) | 0.25 |
| Improved Water Source[30] | 341 | (84.0%) | 32 | (76.2%) | 0.20 |
| Shared Toilet^[[8]](#endnote-8)^ | 200 | (52.4%) | 414 | (45.9%) | 0.03* |
| Toilet Type  Flushing  Pit Latrine  Open Defecation | 29  357  20 | (7.1%)  (87.9%)  (4.9%) | 7  33  0 | (9.7%)  (86.8%)  (0%) | 0.03 |

1. Of those with data available (n = 426) Current breastfeeding for children ≤6 months; breastfeeding practiced when children were under 6 months; n = 2 unknown [↑](#endnote-ref-1)
2. Uninfected, Exposure Status unknown (n = 13), Exposed, infection status unknown (n = 4); Column percentages of children with exposure and infection status known (n = 431) [↑](#endnote-ref-2)
3. Wasted is defined as WHZ < -2 or MUAC <11.5cm while Stunted is determined by HAZ <-2; MUAC is only taken into consideration in children 6 months or older [↑](#endnote-ref-3)
4. Median and interquartile range provided [↑](#endnote-ref-4)
5. Of those with admission and discharge dates both available (n = 443) [↑](#endnote-ref-5)
6. Not mutually exclusive. Total n = 395 (88.2%) received antibiotics, column percentages are of these children. Other antibiotics given: azithromycin (n = 4), co-amoxiclav (n = 1), cefuroxime (n = 5), trimethoprim-sulfamethoxazole (n = 10), chloramphenicol (n = 17), ciprofloxacin (n = 2), clarithromycin (n = 22), erythromycin (n = 2), tetracycline (n = 2), metronidazole (n = 23) [↑](#endnote-ref-6)
7. Not mutually exclusive. Other diagnoses at admission include: HIV (n = 2), urinary tract infection (n = 2), poisoning/herbal toxicity (n = 4), asthma (n = 7), upper respiratory tract infection (n = 31), [↑](#endnote-ref-7)
8. Shared Toilets are those used by more than 1 household and did not include open defecation (n = 20) and excluding those who did not answer (n = 6) [↑](#endnote-ref-8)
